# Prevalence of preeclampsia and the associated risk factors among pregnant women in Bangladesh

**DOI:** 10.1038/s41598-021-00839-w

**Published:** 2021-10-29

**Authors:** Ananya Dutta Mou, Zitu Barman, Mahmudul Hasan, Rakib Miah, Jaasia Momtahena Hafsa, Aporajita Das Trisha, Nurshad Ali

**Affiliations:** Department of Biochemistry and Molecular Biology, Shahjalal University of Science and Technology, Sylhet, 3114 Bangladesh

**Keywords:** Public health, Epidemiology

## Abstract

Preeclampsia is a multi-organ system disorder of pregnancy and is responsible for a significant rate of maternal morbidity and mortality worldwide. In Bangladesh, a large number of obstetric deaths occur every year but the exact reasons are not well investigated. The data regarding preeclampsia and its associated risk factors are scarce or limited in pregnant women in Bangladesh. Therefore, we aimed to conduct a cross-sectional study to estimate the prevalence of preeclampsia and identify the possible risk factors in a pregnant women cohort in Bangladesh. In this cross-sectional study, a total of 111 participants were enrolled and asked to include their anthropometric, socio-demographic, and other related lifestyle information in a standard questionnaire form. Blood samples were also collected from each participant to analyze serum levels of lipid profile, liver enzymes, uric acid, and creatinine by using standard methods. Logistic regression analysis was performed to identify the factors associated with preeclampsia. The overall prevalence of preeclampsia was 14.4%. About 10% of the pregnancies were found to have preeclampsia after 20 weeks of gestation without a previous history of hypertension. On the other hand, the prevalence of preeclampsia that superimposed on chronic hypertension was found to be 5.4%. Serum levels of TC, LDL-C, ALT and uric acid were significantly higher and HDL-C was significantly lower in preeclamptic pregnancies than the non-preeclamptic pregnancies. Respondents who required to take antihypertensive medications (AOR 5.45, 95% CI [1.09, 27.31]) and who never took antenatal care (AOR 6.83, 95% CI [1.00, 46.48]) were more likely to be preeclamptic. In conclusion, the present study showed a comparatively high prevalence of preeclampsia among pregnant women in Bangladesh. Some programmatic interventions such as medication for hypertension, antenatal visits to doctors, delivery and postnatal care services should be considered to reduce and prevent the hypertensive pregnancy disorders in Bangladesh.

## Introduction

Preeclampsia is one of the leading causes of maternal morbidity and mortality in the world^1^. Preeclampsia is hypertension that generally occurs after 20 weeks of gestation along with proteinuria^2^. When proteinuria is absent, preeclampsia is diagnosed in association with liver dysfunction, thrombocytopenia, pulmonary oedema, new onset of kidney dysfunction, or new-onset of cerebral or visual disturbances^3^. It can cause severe morbidity, chronic disability, and even death of mothers and babies. Moreover, it is linked with an increased risk of cardiovascular diseases and type 2 diabetes in a mother’s later life^4^.

In developing countries, women are at 14 times higher risk of dying from obstetric complications compared to developed countries^5^. Pregnancy complications caused around 289,000 deaths of women worldwide in 2013 and 99% of them were from developing countries^5^. Globally, about 12% of mothers die only from preeclampsia^6^. As estimated by WHO, the occurrence of preeclampsia is seven times higher in developing countries compared to developed countries^7^. The prevalence of preeclampsia ranges between 1.8 and 16.7% in developing countries^8^.

Several studies have demonstrated that preeclampsia is linked with the failure of the trophoblastic invasion of maternal spiral arteries which leads to higher vascular resistance of uterine arteries and lower uteroplacental blood flow^9,10^. If unmanaged, preeclampsia can prevent the flow of adequate blood and oxygen to the developing fetus, cause maternal liver and kidney damage and sometimes progress to eclampsia, a serious condition involving seizures^11,12^. There is no known cure for preeclampsia other than the delivery of the baby or the placenta. Early diagnosis and close monitoring can help to control preeclampsia during pregnancy. Developed countries have reduced the incidence of eclampsia and associated mortality by nearly 90% through early detection during antenatal care and by increasing access to hospital care for preeclamptic women^13^. The prevalence of preeclampsia is not well-documented and the associated data are scarce in Bangladesh. Thus, this study aimed to estimate the prevalence of preeclampsia, measure some biochemical variables, and identify the associated risk factors among pregnant women in the Sylhet region of Bangladesh.

## Methods

### Study design and study participants

A cross-sectional study was designed and conducted during the period from October 2019 to October 2020. All the relevant analyses were performed at the Department of Biochemistry and Molecular Biology, Shahjalal University of Science and Technology, Sylhet, Bangladesh. The study participants were enrolled from the Gynae and Obstetrics unit of Sylhet Osmani Medical College, Sylhet Diabetic Hospital, and another private hospital in the Sylhet city area. About 220 pregnant women, who visited these hospitals for their regular checkups, were invited to participate in the study. Among them, 111 women (29 rural and 82 urban) were agreed to participate. Their anthropometric, socio-demographic, individual as well as family history of diabetes and hypertension and lifestyle information were noted in a standard questionnaire form. Information regarding preexisting hypertension and the necessity of antihypertensive medications were either obtained from the participant's self-report or from their medical documents. The anthropometric measurements were made with the assistance of trained medical personnel following the standard procedure described elsewhere^14–19^. Briefly, The systolic blood pressure (SBP) and diastolic blood pressure (DBP) were measured using an automated blood pressure recorder (Omron M10, Japan) while they were seated in a comfortable position after at least 10 min of rest. The weight was recorded to the nearest 0.1 kg (kg) with the subject standing on the weighing machine (Beurer BF 700, Germany) without shoes and wearing light clothing. Inclusion criteria: pregnant women of any trimester were included as study participants. Exclusion criteria: participants suffering from preexisting kidney disease or urinary tract infection, preexisting liver disease, and preexisting thyroid disorder were excluded from the study. During exclusion of the participants, we considered the subject's self-reported evidence and/or medical records of their preexisting diseases.

### Sample collection and biochemical analysis

About 5 ml of the venous blood samples were collected from each participant with the help of expert clinical assistants. Blood samples were then placed on ice and transported immediately to the laboratory. The blood samples were then kept for 10 minutes at room temperature for coagulation and centrifuged (Sorvall ST 8R, Thermo Scientific, Germany) at 4400 rpm for 10 minutes. The isolated serums were then stored in a – 20 °C refrigerator until the biochemical markers were analyzed. Serum levels of TG (triglyceride), TC (total cholesterol), HDL-C (high-density lipoprotein cholesterol), LDL-C (low-density lipoprotein cholesterol), creatinine, and uric acid were measured by colorimetric methods with a biochemistry analyzer (Humalyzer 3000, USA) using available diagnostic kits (Human Diagnostic, Germany)^20–23^. Serum levels of ALT (alanine aminotransferase) and GGT (gamma-glutamyl transferase) were measured by kinetic methods^24^. The proteinuria was determined using the urine dipstick method^5^.

### Diagnostic criteria

Proteinuria: women with a protein level of 1 + were grouped as having proteinuria.

Trimesters, gravidity, and parity: women with gestation periods of week 1 to 12 were included as first trimester, 13 to 28 as second trimester, and 29 to 40 as third trimester. Gravidity was defined as the total number of pregnancies of a woman, regardless of the outcome. Parity was denoted as the number of pregnancies reaching ≥ 24 weeks.

Preeclampsia: Preeclampsia was defined as having hypertension (≥ 140 mmHg SBP and/or ≥ 90 mmHg DBP) along with either proteinuria or elevated liver enzyme (e.g., ALT level > 40 IU/L) or kidney dysfunction (creatinine > 1 mg/dL)^25^. Preeclampsia that developed after 20 weeks of gestation with the previous history of normal blood pressure was denoted as de novo^26^. Preeclampsia with pre-pregnancy hypertension or that developed before 20 weeks of gestation was defined as preeclampsia superimposed on chronic hypertension^27^.

### Data processing and analysis

Descriptive statistics were used to present the baseline data variables. P-values were obtained from the independent sample t-test for comparison between quantitative variables. Binary logistic regression was applied to determine the relationship between the dependent variables and independent variables. The independent variables that were significant at univariate analysis and some relevant variables were included in the multiple logistic regression model. IBM SPSS, version 25.0 was used for statistical data analysis. The p-value < 0.05 was considered statistically significant.

### Ethical consideration

This study was approved by the internal review board at the Department of Biochemistry and Molecular Biology, School of Life Sciences, Shahjalal University of Science and Technology. All methods in the current study were carried out following the relevant guidelines and regulations. All the study participants were informed about the study aims and written informed consent was obtained from them before inclusion in the study.

## Results

### Socio-demographic characteristics of the study participants

A total of 111 pregnant women participated in the study from the Sylhet region. The age range of the subjects was 17 to 37 years, with a mean of 26.4 ± 4.9 years. Overall, the mean of systolic (SBP), diastolic (DBP), and pulse pressure (PP) were 122.2 ± 19.9 mmHg, 79.8 ± 13.6 mmHg, and 42.4 ± 12.0 mmHg, respectively, for the study cohort. Among the study participants, 22.9% had hypertension prior to pregnancy and 19.1% were diabetic, whereas 43.7% of the study subjects had a family history of hypertension, 38.4% had a family history of diabetes and 7% had a family history of hypertensive disorders in pregnancy (Table 1).

**Table 1:** Sociodemographic characteristics of the participants.

Variables	n	%
**Age of respondents**		
< 25	41	36.9
25–29	32	28.8
≥ 30	38	34.2
Mean ± SD (max)	26.4 ± 4.9 (37)	
**Current body weight (Kg)**		
< 65	53	63.9
65–74	16	19.3
≥ 75	14	16.9
Mean ± SD (max)	60.4 ± 11.8 (85)	
SBP (mmHg)	122.2 ± 19.9 (200)	
DBP (mmHg)	79.8 ± 13.6 (150)	
Pulse pressure (mmHg)	42.4 ± 12.0 (80)	
**Living area**		
Rural	29	26.1
Suburban or urban	82	73.9
**Education**		
Primary or elementary	36	34
Secondary	40	37.7
Higher secondary	14	13.2
Graduate or above	16	15.1
**Occupation**		
Jobholder	4	3.7
Student	0	0
Housewife	103	96.3
Pre-pregnancy hypertension	19	22.9
**Requirement of anti-hypertensive medication**		
No	65	77.4
Occasionally or regularly	19	22.6
**Family history of hypertension**		
Yes	38	43.7
No	35	40.2
Don’t know	14	16.1
Diabetes mellitus	18	19.1
**Family history of diabetes mellitus**		
Yes	33	38.4
No	53	61.6
**Family history of hypertensive disorders in pregnancy**		
Yes	3	7.0
No	13	30.2
Don’t know	27	62.8
**Intake of fruits and vegetables**		
Medium to high	72	80.9
Low	17	19.1
**Suffering by**		
Visual problem	15	13.5
Anemia	12	10.8
Minor headache	41	36.9
Nausea	49	44.1
Dizziness	40	36
Chest or back pain	26	23.4
Sleeping problem	32	28.8
Severe headache	18	34
**Frequency of visiting doctor during pregnancy**		
Regularly or occasionally	78	86.7
Never	12	13.3

Information about pre-pregnancy hypertension, diabetes mellitus, and amount of fruits and vegetable intake was collected from the participants. Family: it comprises of a group of kin related to each other by blood connection or by marital tie.

### Obstetrical characteristics of the respondents

Among the total subjects, 19.6% of the participants were in the first trimester, 21.5% in the second trimester, and 58.9% were in the third trimester of their pregnancy. The percentage of primiparous pregnancies was 36.8% in total. 3.4% of the pregnancies were twin in type and the rest 96.6% were singleton. 26.6% of the study subjects were detected as having hypertensive pregnancies. None of the participants had reported convulsion during their pregnancy period (Table 2).

**Table 2:** Obstetrical characteristics of the respondents.

Variables	n	%
**Pregnancy period**		
First trimester	21	19.6
Second trimester	23	21.5
Third trimester	63	58.9
Hypertensive pregnancy	29	26.6
**Primiparous**		
Yes	39	36.8
No	67	63.2
**Pregnancy type**		
Singleton	56	96.6
Twin	2	3.4
**Gravidity**		
1	39	39.8
2	21	21.4
3	21	21.4
≥ 4	17	17.3
**Parity**		
< 2	49	51.0
2 – 3	37	38.5
≥ 4	10	10.4
**Previous stillborn or miscarriage history**		
0	69	74.2
1	20	21.5
≥ 2	4	4.3
**Number of children**		
< 2	78	71.6
≥ 2	31	28.4
Convulsion during pregnancy	0	0

Hypertensive pregnancy: SBP ≥ 140 mmHg and/or DBP ≥ 90 mmHg^25^. Primipara means first pregnancy.

### Prevalence of preeclampsia in study participants

The overall prevalence of preeclampsia was 14% among the study subjects. 9.9% of the pregnancies were found to have preeclampsia after 20 weeks of gestation without prior history of hypertension, whereas, the prevalence of preeclampsia that superimposed on chronic hypertension was found to be 5.4% (Table 3).

**Table 3:** Prevalence of preeclampsia among the study participants.

Categories	n (%)
N	111
Non-preeclamptic pregnancies	92 (83.8)
**Preeclamptic pregnancies**	16 (14.4)
De novo	11 (9.9)
Superimposed on chronic hypertension	6 (5.4)

De novo: preeclampsia with hypertension developed after 20 weeks of gestation^26^. Superimposed: preeclampsia with pre-pregnancy hypertension or that developed before 20 weeks of gestation was defined as preeclampsia superimposed on chronic hypertension^27^.

### Level of blood pressure variables in the study subjects

**Figure 1:**
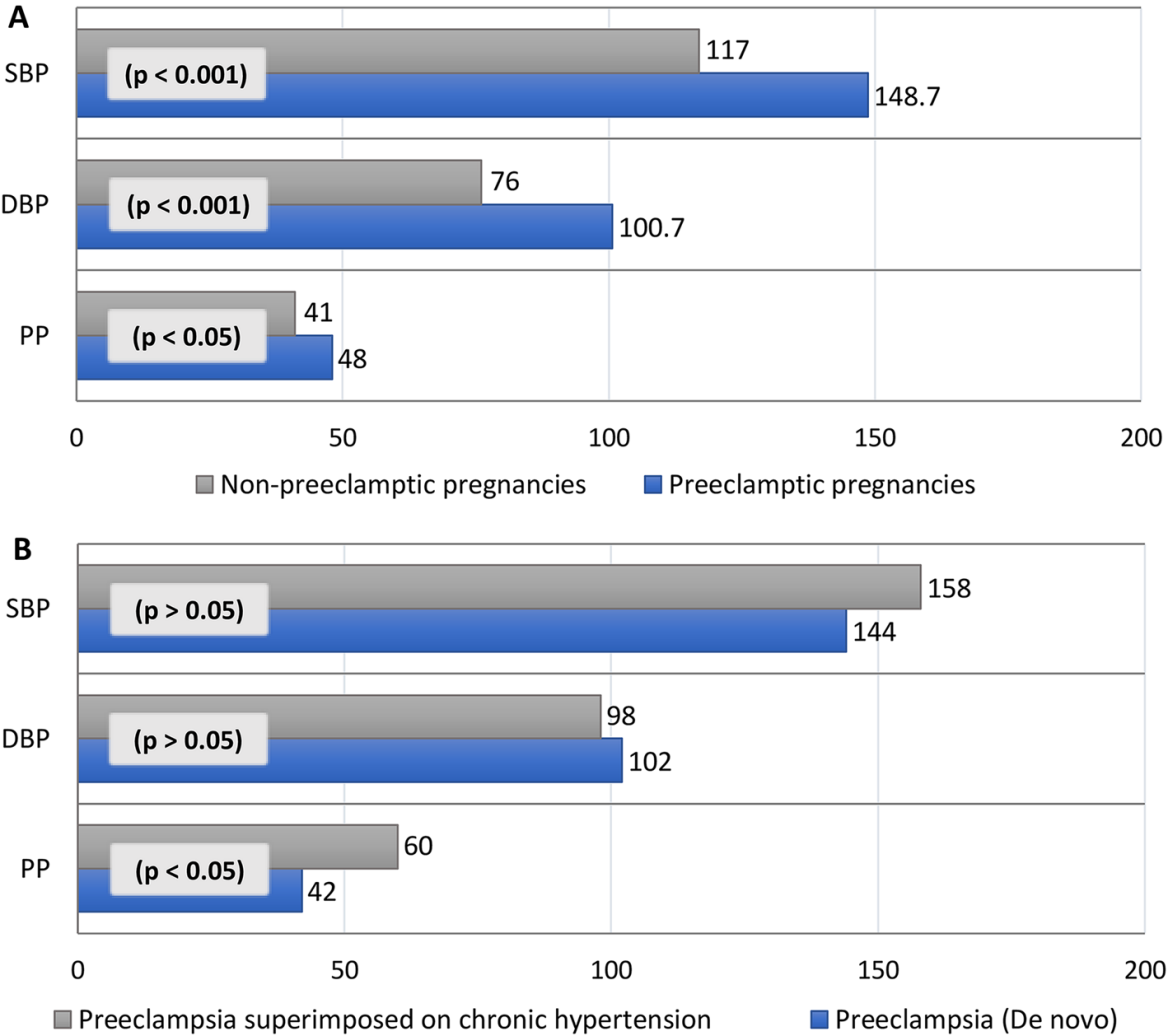
Blood pressure components in preeclamptic and non-preeclamptic pregnancies (**A**); Blood pressure components in pregnancies with preeclampsia and preeclampsia superimposed on chronic hypertension (**B**). Data are presented as mean. *SBP* systolic blood pressure, *DBP* diastolic blood pressure; *PP* pulse pressure (SBP − DBP). P-values are obtained from independent sample t-test in comparison between two groups. De novo: preeclampsia with hypertension developed after 20 weeks of gestation.

Overall, the mean of SBP, DBP, and PP for the preeclamptic pregnancies were 148.7 ± 25.6 mmHg, 100.7 ± 16.2 mmHg, and 48 ± 17.4 mmHg, respectively. All of their values were significantly higher (p < 0.05) in preeclamptic pregnancies compared to non-preeclamptic pregnancies (Fig. 1A). Moreover, PP was found significantly higher in chronic hypertensive pregnancies with preeclampsia compared to de novo appearance after 20 weeks of gestation (Fig. 1B).

### Biochemical markers in preeclamptic and non-preeclamptic pregnancies

Serum levels of TC, LDL-C, ALT and uric acid were significantly higher in preeclamptic pregnancies compared to the pregnancies without preeclampsia (at least p < 0.001 for all cases). The mean level of HDL-C was found significantly lower in preeclamptic subjects than non-preeclamptic subjects (p < 0.01) (Table 4).

**Table 4:** Levels of abnormal biochemical markers in preeclamptic and non-preeclamptic participants.

Variables	Overall	Preeclamptic pregnancy	Non-preeclamptic pregnancy	p-value
TC (mg/dL)	222.9 ± 64.3	276.1 ± 84.8	212.7 ± 56.5	0.001***
TG (mg/dL)	266.4 ± 139.6	310.1 ± 114.8	261.1 ± 145.2	0.234
HDL-C (mg/dL)	28.9 ± 12.5	21.2 ± 7.7	30.7 ± 12.7	0.008**
LDL-C (mg/dL)	139.7 ± 64.6	192.8 ± 72.2	128.5 ± 58.8	0.000***
ALT (U/L)	36.3 ± 27.5	66.4 ± 26.6	30.4 ± 24.0	0.000***
GGT (U/L)	9.5 ± 7.4	7.1 ± 8.8	9.7 ± 7.1	0.299
Uric acid (mg/dL)	4.6 ± 1.4	6.0 ± 1.5	4.4 ± 1.1	0.000***
Creatinine (mg/dL)	0.5 ± 0.21	0.56 ± 0.48	0.54 ± 0.17	0.872

Data are presented as % or mean ± SD. p-values are obtained from Independent sample t-test in comparison between preeclamptic and non-preeclamptic groups. Values greater than the reference range are denoted as “elevated”^44^.

*TC* total cholesterol, *TG* triglyceride, *HDL-C* high density lipoprotein cholesterol, *LDL-C* low density lipoprotein cholesterol, *ALT* alanine aminotransferase, *GGT* gamma glutamyltransferase.

*p < 0.05; **p < 0.01; ***p < 0.001.

### Factors associated with preeclampsia

We also assessed the relationships between socio-demographic factors, obstetrical data, and the prevalence of preeclampsia. In univariate analysis, the requirement of antihypertensive medications, less intake of fruits and vegetables, and not visiting a doctor during pregnancy (antenatal care) were found to be significantly associated with the prevalence of preeclampsia. After adjusting for these significant independent factors using multiple regression analysis, the requirement of antihypertensive medications and lack of antenatal care during pregnancy were found as independent predictors for preeclampsia. Respondents who were required to take antihypertensive medications either occasionally or regularly were more likely to be preeclamptic than those who did not (AOR 5.45, 95% CI [1.09, 27.31]). Women who never consulted any doctor during their pregnancy period had about 6.8 times higher odds of being preeclamptic than women who did occasionally or regularly (AOR 6.83, 95% CI [1.00, 46.48]) (Table 5).

**Table 5:** Multiple logistic regression analysis of factors that can be associated with preeclampsia among pregnant women.

Variables	Preeclampsia		COR (95% CI)	AOR (95% CI)^a^	p-value
	No	Yes			
**Maternal age**					
< 25	34 (85.0)	6 (15.0)	1.71 (0.39–7.43)	0.44 (0.07–2.98)	0.400
25 – 29	29 (90.6)	3 (9.4)	1.00	1.00	
≥ 30	29 (82.9)	6 (17.1)	2.00 (0.46–8.77)	0.92 (0.12–7.15)	0.939
**Body weight (Kg)**					
< 65	47 (92.2)	4 (7.8)	1.00	1.00	
65 – 74	13 (81.3)	3 (18.8)	2.71 (0.54–13.68)	0.88 (0.01–56.16)	0.950
≥ 75	12 (85.7)	2 (14.3)	1.96 (0.32–11.99)	2.19 (0.05–90.22)	0.680
**Pre-pregnancy hypertension**					
No	54 (84.4)	10 (15.6)	1.00	1.00	
Yes	14 (73.7)	5 (26.3)	1.93 (0.57–6.56)	1.03 (0.11–9.28)	0.979
**Requirement of antihypertensive medication**					
Regularly or occasionally	14 (73.7)	5 (26.3)	0.24 (0.06–0.93)*	5.45 (1.09–27.31)	0.039*
No	59 (92.2)	5 (7.8)	1.00	1.00	
**Primiparous**					
Yes	30 (81.1)	7 (18.9)	1.00	1.00	
No	58 (87.9)	8 (12.1)	0.59 (0.20–1.79)	0.57 (0.09–3.46)	0.536
**Gravidity**					
1	30 (81.1)	7 (18.9)	1.40 (0.32–6.11)	1.07 (0.11–10.28)	0.951
2	20 (95.2)	1 (4.8)	0.30 (0.03–3.15)	0.29 (0.02–4.94)	0.391
3	18 (85.7)	3 (14.3)	1.00	1.00	
≥ 4	15 (88.2)	2 (11.8)	0.80 (0.12–5.44)	1.05 (0.11–10.23)	0.968
**Parity**					
< 2	40 (85.1)	7 (14.9)	1.44 (0.39–5.36)	1.57 (0.21–11.55)	0.659
2 – 3	33 (89.2)	4 (10.8)	1.00	1.00	
≥ 4	7 (70.0)	3 (30.0)	3.54 (0.64–19.45)	3.44 (0.34–34.65)	0.294
**Previous stillborn or miscarriage history**					
0	57 (85.1)	10 (14.9)	1.00	1.00	
1	17 (85.0)	3 (15.0)	1.01 (0.25–4.08)	0.77 (0.11–5.39)	0.789
≥ 2	3 (75.0)	1 (25.0)	1.90 (0.18–20.14)	1.83 (0.01–380.63)	0.825
**Number of children**					
< 2	64 (85.3)	11 (14.7)	1.00	1.00	
≥ 2	27 (90.0)	3 (10.0)	0.65 (0.17–2.50)	1.69 (0.24–11.79)	0.596
**Intake of fruits and vegetables**					
Medium to high	65 (90.3)	7 (9.7)	1.00	1.00	
Low	9 (56.3)	7 (43.8)	7.22 (2.05–25.42)**	4.29 (0.84–21.98)	0.081
**Frequency of visiting doctor during pregnancy**					
Regularly or occasionally	68 (88.3)	9 (11.7)	1.00	1.00	
Never	6 (54.5)	5 (45.5)	6.30 (1.59–24.91)**	6.83 (1.00–46.48)	0.050*

*p < 0.05; **p < 0.01.

^a^Adjusted for the variables found significant in univariate regression analysis (Requirement of antihypertensive medication, intake of fruits and vegetables, frequency of visiting doctor during pregnancy).

## Discussion

The present study estimated the prevalence of preeclampsia with some biochemical variables and identified the possible associated risk factors among pregnant women in Bangladesh. The overall prevalence of preeclampsia was 14.4% in pregnancy. Lack of awareness and insufficient antenatal care in rural areas can be the reason behind this higher prevalence.

Up to now, there is limited information on preeclampsia and its associated factors among Bangladeshi women. A recent study reported about 44% preeclampsia among pregnant women who visited a Maternity Clinic in Dhaka, Bangladesh^11^. A probable reason for the higher prevalence of preeclampsia in that study was the inclusion of the participants from their third trimester of pregnancy, whereas in our study we included pregnant women from all three trimesters.

The prevalence of preeclampsia in the present study is higher compared with the global average, which is around 2%^28^. However, the prevalence ranges from 1.8 to 16.7% in developing countries^8^, and thus our finding falls in the upper side of this range. A study conducted in our neighboring country India reported the prevalence of preeclampsia about 28% with a variation in the prevalence across the states or regions^29^. A review covering larger data sets reported the prevalence of preeclampsia 0.2–6.7% in Asia, 0.5–2.3% in Africa, 2.8–5.2% in Europe, 2.8–9.2% in Oceania, 1.8–7.7% in South America and the Caribbean, and 2.6–4.0% in North America^30^. Another review summarized the data for the Asian regions and reported the prevalence of preeclampsia 2.07% in China, 1.19% in Japan, 2.22% in Thailand and 0.59% in Nepal^31^. Some possible reasons may be associated with the high prevalence of preeclampsia in Bangladeshi pregnant women compared to pregnant women from other countries. For example, a large proportion of our study participants were in their third trimester of pregnancy which can be a possible underlying factor of higher prevalence of preeclampsia. Moreover, Bangladesh is a developing country with lower health concerns in the overall population especially people living in the rural regions. Furthermore, socioeconomic status and not taking treatment at the proper time may also be associated with the high prevalence of preeclampsia in Bangladeshi pregnant women.

In the present study, we also measured some biochemical variables and we observed that participants with preeclampsia had significantly increased serum levels of TC, and LDL-C and significantly decreased levels of HDL-C compared to subjects with normal pregnancy (p < 0.01), which is in line with a study finding conducted by Wang et al.^32^. Moreover, we found a significantly increased serum level of ALT in preeclamptic pregnancies compared to normal pregnant women. The high level of ALT may indicate liver dysfunction due to preeclamptic damage. In a previous study, serum ALT level was found significantly higher in preeclampsia/eclampsia groups compared to women with normal pregnancies^33^. An elevated level of ALT was also observed in other studies which are in line with our present study^34,35^.

In this study, serum uric acid levels were significantly higher in preeclamptic pregnancies compared to non-preeclamptic pregnancies, which is similar to other reports^33,36^. Hyperuricemia is now considered one of the initial laboratory manifestations of preeclampsia^36,37^. Several studies have demonstrated a positive relationship between serum uric acid and hypertension in the general population and pregnant women^19,38^. Thus, monitoring of serum uric acid levels in the pregnancy period might be helpful in early detection and intervention of preeclampsia and in preventing associated complications.

In the present study, we identified some possible risk factors that are associated with preeclamptic pregnancies. For example, we found that women who are required to take antihypertensive medications for controlling their blood pressure level are more likely to suffer from preeclampsia. Additionally, women who consulted doctors regularly or occasionally during their pregnancy were less affected by preeclampsia compared to the women who never visited the doctor. Our finding is similar to a study conducted by Uddin et al. which showed that women having poor access to antenatal care were more affected by hypertension in their pregnancy^39^. According to a report, about 64% of pregnant women in Bangladesh aged 15–49 receive at least one antenatal care visit with a skilled health provider^40^. The detection and counselling of preeclampsia essentially depend on the frequency of antenatal care and measurement of the blood pressure of the mother during the visit. In developing countries, many women with preeclampsia, mostly at the community level, still do not get proper antenatal care and are at higher risk of developing serious complications.

A recent study determined the possible risk factors for preeclampsia in pregnant women who went to a tertiary hospital in Dhaka, Bangladesh showed that education level, occupation, bad obstetrics history, or history of abortion were not significantly associated with preeclampsia development^41^, which is similar to our study findings. According to the same study, preeclampsia was high in women having poor antenatal care, which is also in line with ours. Although obesity, primigravida, preexisting hypertension, advanced maternal age, and family history of hypertension are common risk factors for preeclampsia globally^42,43^, we did not find any of them significantly associated with preeclampsia. This variation of our study with other studies may be a result of the relatively small number of participants of our study enrolled from a single region of Bangladesh.

Although the present study estimated the prevalence of preeclampsia and identified some associated risk factors, the study also had some limitations. Firstly, the study participants were included in this study from hospitals and healthcare centers which may increase the prevalence of preeclampsia. Secondly, we could not collect detailed information on participants’ access to hospital care facilities which should be considered in such a study. Thirdly, the sample size in our study was relatively small and the study was conducted in a divisional region of Bangladesh, thus our findings may not be nationally representative. However, our findings may be useful for further studies in this area in Bangladesh.

## Conclusion

The prevalence of preeclampsia was comparatively higher in rural areas of Bangladesh. Serum levels of TC, LDL-C, ALT and uric acid were found to be significantly higher and HDL-C was significantly lower in preeclamptic pregnancies compared to the pregnancies without preeclampsia. Participants who were required to take antihypertensive medications either occasionally or regularly and who never took antenatal care were found more likely to have preeclampsia. Thus, the presence of preeclampsia can also be predicted by investigating some biochemical components and proper antenatal care is an important part of prevention. Furthermore, prospective studies are needed to understand the underlying mechanisms of preeclampsia in pregnancies and their proper management strategies.
